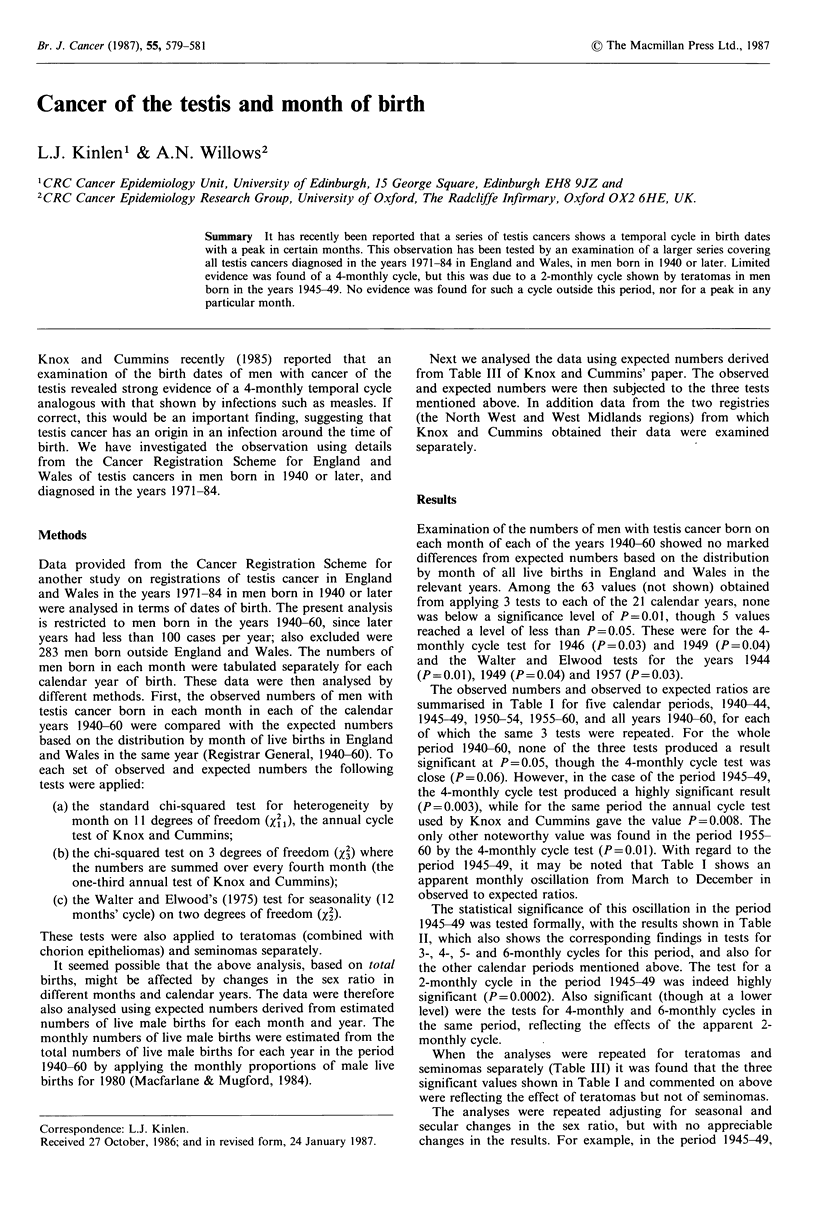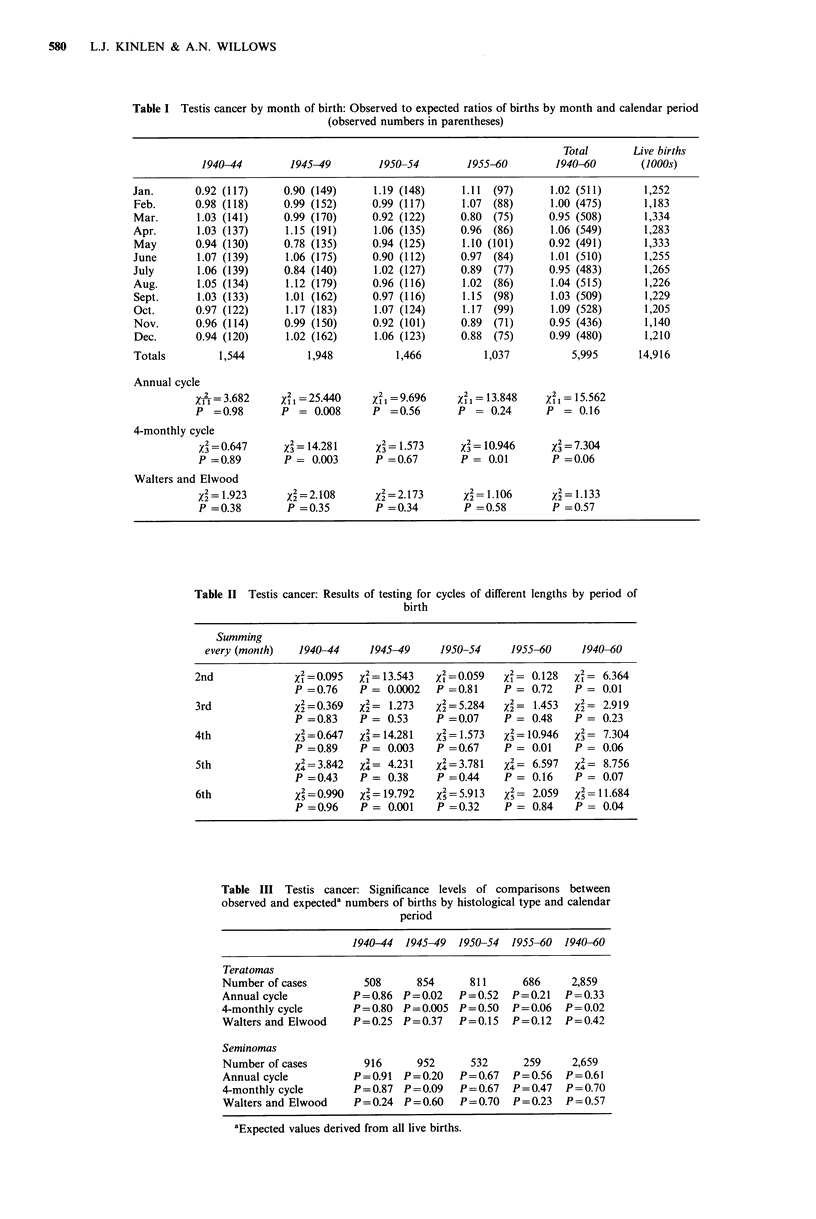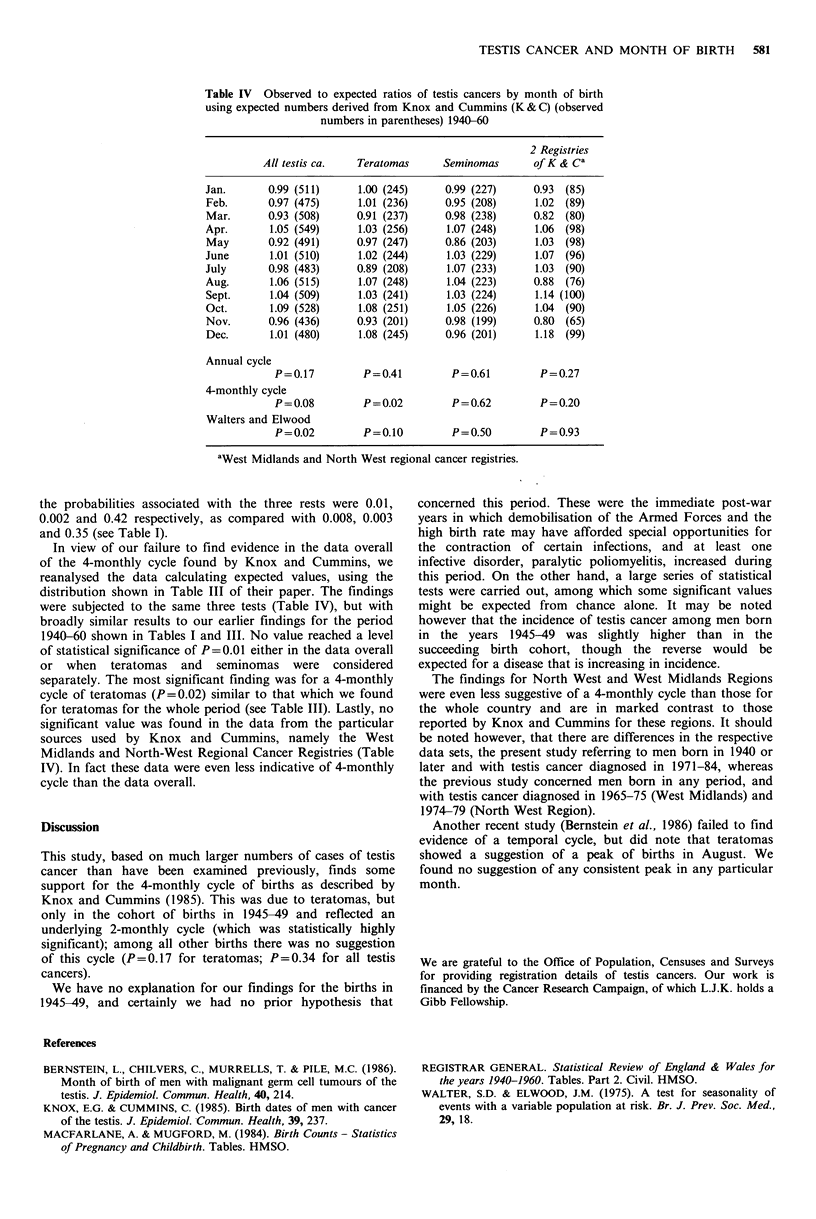# Cancer of the testis and month of birth.

**DOI:** 10.1038/bjc.1987.117

**Published:** 1987-05

**Authors:** L. J. Kinlen, A. N. Willows

## Abstract

It has recently been reported that a series of testis cancers shows a temporal cycle in birth dates with a peak in certain months. This observation has been tested by an examination of a larger series covering all testis cancers diagnosed in the years 1971-84 in England and Wales, in men born in 1940 or later. Limited evidence was found of a 4-monthly cycle, but this was due to a 2-monthly cycle shown by teratomas in men born in the years 1945-49. No evidence was found for such a cycle outside this period, nor for a peak in any particular month.


*Br. J. Cancer* (1987), **55**, 579–581

© The Macmillan Press Ltd., 1987

### Cancer of the testis and month of birth

L.J. Kinlen[1] & A.N. Willows[2]

[1]*CRC Cancer Epidemiology Unit, University of Edinburgh, 15 George Square, Edinburgh EH8 9JZ and*
[2]*CRC Cancer Epidemiology Research Group, University of Oxford, The Radcliffe Infirmary, Oxford OX2 6HE, UK.*

**Summary** It has recently been reported that a series of testis cancers shows a temporal cycle in birth dates with a peak in certain months. This observation has been tested by an examination of a larger series covering all testis cancers diagnosed in the years 1971–84 in England and Wales, in men born in 1940 or later. Limited evidence was found of a 4-monthly cycle, but this was due to a 2-monthly cycle shown by teratomas in men born in the years 1945–49. No evidence was found for such a cycle outside this period, nor for a peak in any particular month.

Knox and Cummins recently (1985) reported that an examination of the birth dates of men with cancer of the testis revealed strong evidence of a 4-monthly temporal cycle analogous with that shown by infections such as measles. If correct, this would be an important finding, suggesting that testis cancer has an origin in an infection around the time of birth. We have investigated the observation using details from the Cancer Registration Scheme for England and Wales of testis cancers in men born in 1940 or later, and diagnosed in the years 1971–84.

### Methods

Data provided from the Cancer Registration Scheme for another study on registrations of testis cancer in England and Wales in the years 1971–84 in men born in 1940 or later were analysed in terms of dates of birth. The present analysis is restricted to men born in the years 1940–60, since later years had less than 100 cases per year; also excluded were 283 men born outside England and Wales. The numbers of men born in each month were tabulated separately for each calendar year of birth. These data were then analysed by different methods. First, the observed numbers of men with testis cancer born in each month in each of the calendar years 1940–60 were compared with the expected numbers based on the distribution by month of live births in England and Wales in the same year (Registrar General, 1940–60). To each set of observed and expected numbers the following tests were applied:

(a) the standard chi-squared test for heterogeneity by month on 11 degrees of freedom ($\chi^2_{11}$), the annual cycle test of Knox and Cummins;
(b) the chi-squared test on 3 degrees of freedom ($\chi^2_3$) where the numbers are summed over every fourth month (the one-third annual test of Knox and Cummins);
(c) the Walter and Elwood's (1975) test for seasonality (12 months' cycle) on two degrees of freedom ($\chi^2_2$).

These tests were also applied to teratomas (combined with chorion epitheliomas) and seminomas separately.

It seemed possible that the above analysis, based on *total* births, might be affected by changes in the sex ratio in different months and calendar years. The data were therefore also analysed using expected numbers derived from estimated numbers of live male births for each month and year. The monthly numbers of live male births were estimated from the total numbers of live male births for each year in the period 1940–60 by applying the monthly proportions of male live births for 1980 (Macfarlane & Mugford, 1984).

Correspondence: L.J. Kinlen.
Received 27 October, 1986; and in revised form, 24 January 1987.

Next we analysed the data using expected numbers derived from Table III of Knox and Cummins' paper. The observed and expected numbers were then subjected to the three tests mentioned above. In addition data from the two registries (the North West and West Midlands regions) from which Knox and Cummins obtained their data were examined separately.

### Results

Examination of the numbers of men with testis cancer born on each month of each of the years 1940–60 showed no marked differences from expected numbers based on the distribution by month of all live births in England and Wales in the relevant years. Among the 63 values (not shown) obtained from applying 3 tests to each of the 21 calendar years, none was below a significance level of $P=0.01$, though 5 values reached a level of less than $P=0.05$. These were for the 4-monthly cycle test for 1946 ($P=0.03$) and 1949 ($P=0.04$) and the Walter and Elwood tests for the years 1944 ($P=0.01$), 1949 ($P=0.04$) and 1957 ($P=0.03$).

The observed numbers and observed to expected ratios are summarised in Table I for five calendar periods, 1940–44, 1945–49, 1950–54, 1955–60, and all years 1940–60, for each of which the same 3 tests were repeated. For the whole period 1940–60, none of the three tests produced a result significant at $P=0.05$, though the 4-monthly cycle test was close ($P=0.06$). However, in the case of the period 1945–49, the 4-monthly cycle test produced a highly significant result ($P=0.003$), while for the same period the annual cycle test used by Knox and Cummins gave the value $P=0.008$. The only other noteworthy value was found in the period 1955–60 by the 4-monthly cycle test ($P=0.01$). With regard to the period 1945–49, it may be noted that Table I shows an apparent monthly oscillation from March to December in observed to expected ratios.

The statistical significance of this oscillation in the period 1945–49 was tested formally, with the results shown in Table II, which also shows the corresponding findings in tests for 3-, 4-, 5- and 6-monthly cycles for this period, and also for the other calendar periods mentioned above. The test for a 2-monthly cycle in the period 1945–49 was indeed highly significant ($P=0.0002$). Also significant (though at a lower level) were the tests for 4-monthly and 6-monthly cycles in the same period, reflecting the effects of the apparent 2-monthly cycle.

When the analyses were repeated for teratomas and seminomas separately (Table III) it was found that the three significant values shown in Table I and commented on above were reflecting the effect of teratomas but not of seminomas.

The analyses were repeated adjusting for seasonal and secular changes in the sex ratio, but with no appreciable changes in the results. For example, in the period 1945–49,

**Table I** Testis cancer by month of birth: Observed to expected ratios of births by month and calendar period (observed numbers in parentheses)

| | *1940–44* | *1945–49* | *1950–54* | *1955–60* | *Total 1940–60* | *Live births (1000s)* |
|---|---|---|---|---|---|---|
| Jan. | 0.92 (117) | 0.90 (149) | 1.19 (148) | 1.11 (97) | 1.02 (511) | 1,252 |
| Feb. | 0.98 (118) | 0.99 (152) | 0.99 (117) | 1.07 (88) | 1.00 (475) | 1,183 |
| Mar. | 1.03 (141) | 0.99 (170) | 0.92 (122) | 0.80 (75) | 0.95 (508) | 1,334 |
| Apr. | 1.03 (137) | 1.15 (191) | 1.06 (135) | 0.96 (86) | 1.06 (549) | 1,283 |
| May | 0.94 (130) | 0.78 (135) | 0.94 (125) | 1.10 (101) | 0.92 (491) | 1,333 |
| June | 1.07 (139) | 1.06 (175) | 0.90 (112) | 0.97 (84) | 1.01 (510) | 1,255 |
| July | 1.06 (139) | 0.84 (140) | 1.02 (127) | 0.89 (77) | 0.95 (483) | 1,265 |
| Aug. | 1.05 (134) | 1.12 (179) | 0.96 (116) | 1.02 (86) | 1.04 (515) | 1,226 |
| Sept. | 1.03 (133) | 1.01 (162) | 0.97 (116) | 1.15 (98) | 1.03 (509) | 1,229 |
| Oct. | 0.97 (122) | 1.17 (183) | 1.07 (124) | 1.17 (99) | 1.09 (528) | 1,205 |
| Nov. | 0.96 (114) | 0.99 (150) | 0.92 (101) | 0.89 (71) | 0.95 (436) | 1,140 |
| Dec. | 0.94 (120) | 1.02 (162) | 1.06 (123) | 0.88 (75) | 0.99 (480) | 1,210 |
| Totals | 1,544 | 1,948 | 1,466 | 1,037 | 5,995 | 14,916 |
| Annual cycle | | | | | | |
| | $\chi^2_{11}=3.682$ $P=0.98$ | $\chi^2_{11}=25.440$ $P=0.008$ | $\chi^2_{11}=9.696$ $P=0.56$ | $\chi^2_{11}=13.848$ $P=0.24$ | $\chi^2_{11}=15.562$ $P=0.16$ | |
| 4-monthly cycle | | | | | | |
| | $\chi^2_3=0.647$ $P=0.89$ | $\chi^2_3=14.281$ $P=0.003$ | $\chi^2_3=1.573$ $P=0.67$ | $\chi^2_3=10.946$ $P=0.01$ | $\chi^2_3=7.304$ $P=0.06$ | |
| Walters and Elwood | | | | | | |
| | $\chi^2_2=1.923$ $P=0.38$ | $\chi^2_2=2.108$ $P=0.35$ | $\chi^2_2=2.173$ $P=0.34$ | $\chi^2_2=1.106$ $P=0.58$ | $\chi^2_2=1.133$ $P=0.57$ | |

**Table II** Testis cancer: Results of testing for cycles of different lengths by period of birth

| *Summing every (month)* | *1940–44* | *1945–49* | *1950–54* | *1955–60* | *1940–60* |
|---|---|---|---|---|---|
| 2nd | $\chi^2_1=0.095$ $P=0.76$ | $\chi^2_1=13.543$ $P=0.0002$ | $\chi^2_1=0.059$ $P=0.81$ | $\chi^2_1=0.128$ $P=0.72$ | $\chi^2_1=6.364$ $P=0.01$ |
| 3rd | $\chi^2_2=0.369$ $P=0.83$ | $\chi^2_2=1.273$ $P=0.53$ | $\chi^2_2=5.284$ $P=0.07$ | $\chi^2_2=1.453$ $P=0.48$ | $\chi^2_2=2.919$ $P=0.23$ |
| 4th | $\chi^2_3=0.647$ $P=0.89$ | $\chi^2_3=14.281$ $P=0.003$ | $\chi^2_3=1.573$ $P=0.67$ | $\chi^2_3=10.946$ $P=0.01$ | $\chi^2_3=7.304$ $P=0.06$ |
| 5th | $\chi^2_4=3.842$ $P=0.43$ | $\chi^2_4=4.231$ $P=0.38$ | $\chi^2_4=3.781$ $P=0.44$ | $\chi^2_4=6.597$ $P=0.16$ | $\chi^2_4=8.756$ $P=0.07$ |
| 6th | $\chi^2_5=0.990$ $P=0.96$ | $\chi^2_5=19.792$ $P=0.001$ | $\chi^2_5=5.913$ $P=0.32$ | $\chi^2_5=2.059$ $P=0.84$ | $\chi^2_5=11.684$ $P=0.04$ |

**Table III** Testis cancer: Significance levels of comparisons between observed and expected[a] numbers of births by histological type and calendar period

| | *1940–44* | *1945–49* | *1950–54* | *1955–60* | *1940–60* |
|---|---|---|---|---|---|
| *Teratomas* | | | | | |
| Number of cases | 508 | 854 | 811 | 686 | 2,859 |
| Annual cycle | $P=0.86$ | $P=0.02$ | $P=0.52$ | $P=0.21$ | $P=0.33$ |
| 4-monthly cycle | $P=0.80$ | $P=0.005$ | $P=0.50$ | $P=0.06$ | $P=0.02$ |
| Walters and Elwood | $P=0.25$ | $P=0.37$ | $P=0.15$ | $P=0.12$ | $P=0.42$ |
| *Seminomas* | | | | | |
| Number of cases | 916 | 952 | 532 | 259 | 2,659 |
| Annual cycle | $P=0.91$ | $P=0.20$ | $P=0.67$ | $P=0.56$ | $P=0.61$ |
| 4-monthly cycle | $P=0.87$ | $P=0.09$ | $P=0.67$ | $P=0.47$ | $P=0.70$ |
| Walters and Elwood | $P=0.24$ | $P=0.60$ | $P=0.70$ | $P=0.23$ | $P=0.57$ |

[a]Expected values derived from all live births.

**Table IV** Observed to expected ratios of testis cancers by month of birth using expected numbers derived from Knox and Cummins (K & C) (observed numbers in parentheses) 1940–60

| | *All testis ca.* | *Teratomas* | *Seminomas* | *2 Registries of K & C*[a] |
|---|---|---|---|---|
| Jan. | 0.99 (511) | 1.00 (245) | 0.99 (227) | 0.93 (85) |
| Feb. | 0.97 (475) | 1.01 (236) | 0.95 (208) | 1.02 (89) |
| Mar. | 0.93 (508) | 0.91 (237) | 0.98 (238) | 0.82 (80) |
| Apr. | 1.05 (549) | 1.03 (256) | 1.07 (248) | 1.06 (98) |
| May | 0.92 (491) | 0.97 (247) | 0.86 (203) | 1.03 (98) |
| June | 1.01 (510) | 1.02 (244) | 1.03 (229) | 1.07 (96) |
| July | 0.98 (483) | 0.89 (208) | 1.07 (233) | 1.03 (90) |
| Aug. | 1.06 (515) | 1.07 (248) | 1.04 (223) | 0.88 (76) |
| Sept. | 1.04 (509) | 1.03 (241) | 1.03 (224) | 1.14 (100) |
| Oct. | 1.09 (528) | 1.08 (251) | 1.05 (226) | 1.04 (90) |
| Nov. | 0.96 (436) | 0.93 (201) | 0.98 (199) | 0.80 (65) |
| Dec. | 1.01 (480) | 1.08 (245) | 0.96 (201) | 1.18 (99) |
| Annual cycle | | | | |
| | $P=0.17$ | $P=0.41$ | $P=0.61$ | $P=0.27$ |
| 4-monthly cycle | | | | |
| | $P=0.08$ | $P=0.02$ | $P=0.62$ | $P=0.20$ |
| Walters and Elwood | | | | |
| | $P=0.02$ | $P=0.10$ | $P=0.50$ | $P=0.93$ |

[a]West Midlands and North West regional cancer registries.

the probabilities associated with the three rests were 0.01, 0.002 and 0.42 respectively, as compared with 0.008, 0.003 and 0.35 (see Table I).

In view of our failure to find evidence in the data overall of the 4-monthly cycle found by Knox and Cummins, we reanalysed the data calculating expected values, using the distribution shown in Table III of their paper. The findings were subjected to the same three tests (Table IV), but with broadly similar results to our earlier findings for the period 1940–60 shown in Tables I and III. No value reached a level of statistical significance of $P=0.01$ either in the data overall or when teratomas and seminomas were considered separately. The most significant finding was for a 4-monthly cycle of teratomas ($P=0.02$) similar to that which we found for teratomas for the whole period (see Table III). Lastly, no significant value was found in the data from the particular sources used by Knox and Cummins, namely the West Midlands and North-West Regional Cancer Registries (Table IV). In fact these data were even less indicative of 4-monthly cycle than the data overall.

### Discussion

This study, based on much larger numbers of cases of testis cancer than have been examined previously, finds some support for the 4-monthly cycle of births as described by Knox and Cummins (1985). This was due to teratomas, but only in the cohort of births in 1945–49 and reflected an underlying 2-monthly cycle (which was statistically highly significant); among all other births there was no suggestion of this cycle ($P=0.17$ for teratomas; $P=0.34$ for all testis cancers).

We have no explanation for our findings for the births in 1945–49, and certainly we had no prior hypothesis that concerned this period. These were the immediate post-war years in which demobilisation of the Armed Forces and the high birth rate may have afforded special opportunities for the contraction of certain infections, and at least one infective disorder, paralytic poliomyelitis, increased during this period. On the other hand, a large series of statistical tests were carried out, among which some significant values might be expected from chance alone. It may be noted however that the incidence of testis cancer among men born in the years 1945–49 was slightly higher than in the succeeding birth cohort, though the reverse would be expected for a disease that is increasing in incidence.

The findings for North West and West Midlands Regions were even less suggestive of a 4-monthly cycle than those for the whole country and are in marked contrast to those reported by Knox and Cummins for these regions. It should be noted however, that there are differences in the respective data sets, the present study referring to men born in 1940 or later and with testis cancer diagnosed in 1971–84, whereas the previous study concerned men born in any period, and with testis cancer diagnosed in 1965–75 (West Midlands) and 1974–79 (North West Region).

Another recent study (Bernstein *et al.*, 1986) failed to find evidence of a temporal cycle, but did note that teratomas showed a suggestion of a peak of births in August. We found no suggestion of any consistent peak in any particular month.

We are grateful to the Office of Population, Censuses and Surveys for providing registration details of testis cancers. Our work is financed by the Cancer Research Campaign, of which L.J.K. holds a Gibb Fellowship.

### References

BERNSTEIN, L., CHILVERS, C., MURRELLS, T. & PILE, M.C. (1986). Month of birth of men with malignant germ cell tumours of the testis. *J. Epidemiol. Commun. Health*, **40**, 214.

KNOX, E.G. & CUMMINS, C. (1985). Birth dates of men with cancer of the testis. *J. Epidemiol. Commun. Health*, **39**, 237.

MACFARLANE, A. & MUGFORD, M. (1984). *Birth Counts – Statistics of Pregnancy and Childbirth*. Tables. HMSO.

REGISTRAR GENERAL. *Statistical Review of England & Wales for the years 1940–1960*. Tables. Part 2. Civil. HMSO.

WALTER, S.D. & ELWOOD, J.M. (1975). A test for seasonality of events with a variable population at risk. *Br. J. Prev. Soc. Med.*, **29**, 18.